# Immunomodulatory Prodrug Micelles Imitate Mild Heat Effects to Reshape Tumor Microenvironment for Enhanced Cancer Immunotherapy

**DOI:** 10.1021/acsnano.3c11186

**Published:** 2024-02-12

**Authors:** Thi-Lan-Huong Ngo, Kuan-Lin Wang, Wen-Yu Pan, Ting Ruan, Yu-Jung Lin

**Affiliations:** † Research Center for Applied Sciences, Academia Sinica, Taipei, 115201, Taiwan; ‡ School of Medicine, College of Medicine, Fu Jen Catholic University, New Taipei City, 242062, Taiwan; § School of Medical Laboratory Science and Biotechnology, College of Medical Science and Technology, Taipei Medical University, Taipei, 110301, Taiwan; ∥ Ph.D. Program in Medical Biotechnology, College of Medical Science and Technology, Taipei Medical University, Taipei, 110301, Taiwan

**Keywords:** tumor microenvironment, immune checkpoint inhibitor, heat sensor, TRPV1, capsaicin, immunomodulation

## Abstract

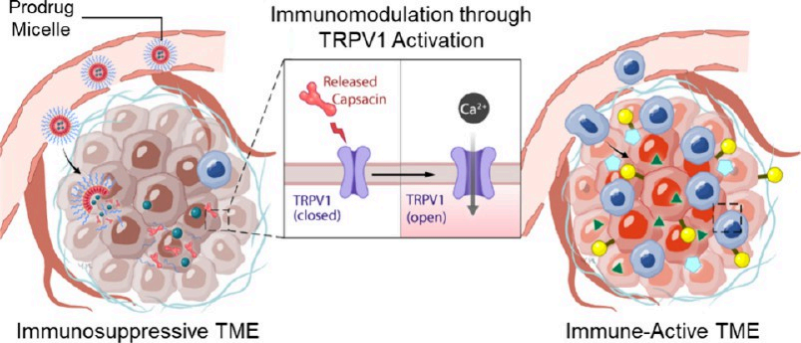

Physical stimulation with mild heat possesses the notable ability to induce immunomodulation within the tumor microenvironment (TME). It transforms the immunosuppressive TME into an immune-active state, making tumors more receptive to immune checkpoint inhibitor (ICI) therapy. Transient receptor potential vanilloid 1 (TRPV1), which can be activated by mild heat, holds the potential to induce these alterations in the TME. However, achieving precise temperature control within tumors while protecting neighboring tissues remains a significant challenge when using external heat sources. Taking inspiration from the heat sensation elicited by capsaicin-containing products activating TRPV1, this study employs capsaicin to chemically stimulate TRPV1, imitating immunomodulatory benefits akin to those induced by mild heat. This involves developing a glutathione (GSH)-responsive immunomodulatory prodrug micelle system to deliver capsaicin and an ICI (BMS202) concurrently. Following intravenous administration, the prodrug micelles accumulate at the tumor site through the enhanced permeability and retention effect. Within the GSH-rich TME, the micelles disintegrate and release capsaicin and BMS202. The released capsaicin activates TRPV1 expressed in the TME, enhancing programmed death ligand 1 expression on tumor cell surfaces and promoting T cell recruitment into the TME, rendering it more immunologically active. Meanwhile, the liberated BMS202 blocks immune checkpoints on tumor cells and T cells, activating the recruited T cells and ultimately eradicating the tumors. This innovative strategy represents a comprehensive approach to fine-tune the TME, significantly amplifying the effectiveness of cancer immunotherapy by exploiting the TRPV1 pathway and enabling *in situ* control of immunomodulation within the TME.

Immune checkpoints, such as programmed cell death 1 (PD-1), are expressed on the surfaces of immune cells, especially T cells. They interact with their partner proteins, like programmed death ligand 1 (PD-L1), typically found on normal cells. This interaction sends an inhibitory signal to T cells, dampening their activation, which is essential for preventing unnecessary immune attacks and maintaining immune hemostasis in normal conditions.^1,2^ However, tumor cells can exploit this mechanism by abnormally upregulating the expression of PD-L1 on their surfaces, allowing them to evade T cell immune surveillance and thrive unchecked.^3−5^

To address this challenge, significant advancements have been made with the development of immune checkpoint inhibitors (ICIs). These inhibitors effectively block the interaction between immune checkpoints on T cells and tumor cells, promoting T cell-mediated immune responses that eliminate tumor cells.^2,3,6−8^ Despite these advances, a noteworthy fraction of tumors is still unlikely to respond to the immunotherapeutic effects of ICIs. This is due to the presence of an immunosuppressive (or immunologically cold) tumor microenvironment (TME), leading to inadequate stimulation of the immune system.^8^

Within the immunosuppressive TME, cold tumor cells display restricted PD-L1 expression on their surfaces and lack T cell infiltration, leading to poor responses to ICI treatments.^8,9^ Conversely, within the immune-active TME, hot tumor cells demonstrate heightened surface PD-L1 expression and are accompanied by abundant T cell infiltration.^8^ Once activated, these T cells can secrete elevated levels of granzyme B (GrB) and proinflammatory cytokines, including interferon γ (IFN-γ), interleukin-2 (IL-2), and tumor necrosis factor α (TNF-α). The secreted molecules not only inhibit tumor growth but also attract more T cells to the TME.^10^ Consequently, hot tumors demonstrate more favorable responses to PD-1/PD-L1 blockade therapy. Therefore, an important strategy for successful PD-1/PD-L1 blockade treatment lies in transforming the TME from immunosuppressive to immune-active, which involves increasing the expression of PD-L1 on tumor cells and enhancing the recruitment and activation of T cells.^8,9^

Significant efforts have been directed toward investigating factors that might restructure the TME into a more immune-active environment, including approaches that involve increasing its temperature.^11−13^ While intense heat generated through thermal ablation directly eliminates tumors at temperatures exceeding 50 °C,^14,15^ the application of mild heat, which raises the TME temperature in a range of 43–45 °C, is poised to reshape the immune landscape within the TME.^8,16^ This subtle temperature increase triggers an upregulation of PD-L1 expression on tumor cells and facilitates the recruitment of T cells into the TME, transforming it into an immune-active state and rendering it more responsive to ICI treatments. As a result, the ICI induces the activation of T cells, amplifying local proinflammatory cytokine levels, ultimately intensifying the eradication of tumor cells.^8,16^ Nevertheless, the precise mechanism driving the immunomodulation induced by mild heat in the TME remains to be understood.^8^

Research has indicated that mild heat, approximately at 43 °C, can activate transient receptor potential vanilloid 1 (TRPV1).^17,18^ Upon physical stimulation by mild heat, TRPV1 initiates calcium signaling in the immune cells and triggers an increase in the secretion of proinflammatory cytokines, leading to the development of an inflamed condition within the TME.^18^ Hence, the activation of TRPV1 has the potential to induce immunomodulation within the TME in response to mild heat, fostering an immunologically active TME. However, achieving precise temperature control within the specific range (43–45 °C) for the tumor while ensuring the safety of neighboring tissues poses a significant challenge when utilizing external approaches.

Drawing from common experiences, the application of products containing capsaicin, a pungent chemical compound derived from chili peppers, frequently triggers a sensation of heat. This phenomenon is linked to the activation of TRPV1.^19^ Given the ability of capsaicin to activate TRPV1, this study proposes an intriguing hypothesis: replacing mild heat with chemical stimulation through capsaicin to activate TRPV1 within the TME, thereby replicating similar immunomodulatory benefits as those generated by mild heat. Unlike physical stimulation with mild heat, utilizing capsaicin for chemical stimulation offers the advantage of *in situ* controlling TRPV1 activation without causing temperature elevation. However, the practical implementation of capsaicin encounters obstacles, such as its poor aqueous solubility (13 μg/mL) and potential chemical toxicity, particularly at higher dosages.^20^

Taking these factors into account, a glutathione (GSH)-responsive immunomodulatory prodrug micelle system is developed in the study. This system has the capability to codeliver a TRPV1 chemical activator (capsaicin) and an ICI (BMS202), with the purpose of addressing aforementioned challenges. While anti-PD-1 and anti-PD-L1 monoclonal antibodies are commonly used in ICI therapy, certain shortcomings persist, including immune-mediated pneumonitis, thyroiditis, and hepatitis.^21^ Given these concerns, BMS202, a hydrophobic small-molecule ICI that has captured great research interest recently due to its notable stability and minimal immunogenicity,^22−25^ is employed in this study. An important strategy introduced here involves the utilization of a GSH-responsive capsaicin prodrug. Prodrugs are known for their capacity to respond to the TME and release bioactive compounds accurately at tumor sites, aiming to minimize systemic toxicity and the occurrence of adverse events.^26^

Figure 1 provides a clear visual representation of the immunomodulatory prodrug micelle system (BMS202-loaded micelle), showcasing its composition and structure, its response to GSH, along with its functional mechanism for transforming the immunosuppressive TME into an immune-active environment. This transformation enhances the effectiveness of ICI therapy. In the prodrug synthesis process, a hydrophobic TRPV1 chemical activator (capsaicin) is linked to a hydrophilic and biocompatible polymer, methoxypolyethylene glycol (mPEG), through a disulfide-containing linker known as 3,3-dithiodipropionic acid (DTPA), which is responsive to GSH.^27^ In an aqueous environment, the as-synthesized amphiphilic capsaicin prodrug self-assembles into micelles, encapsulating BMS202 within their hydrophobic cores to form the BMS202-loaded micelles.

**Figure 1:**
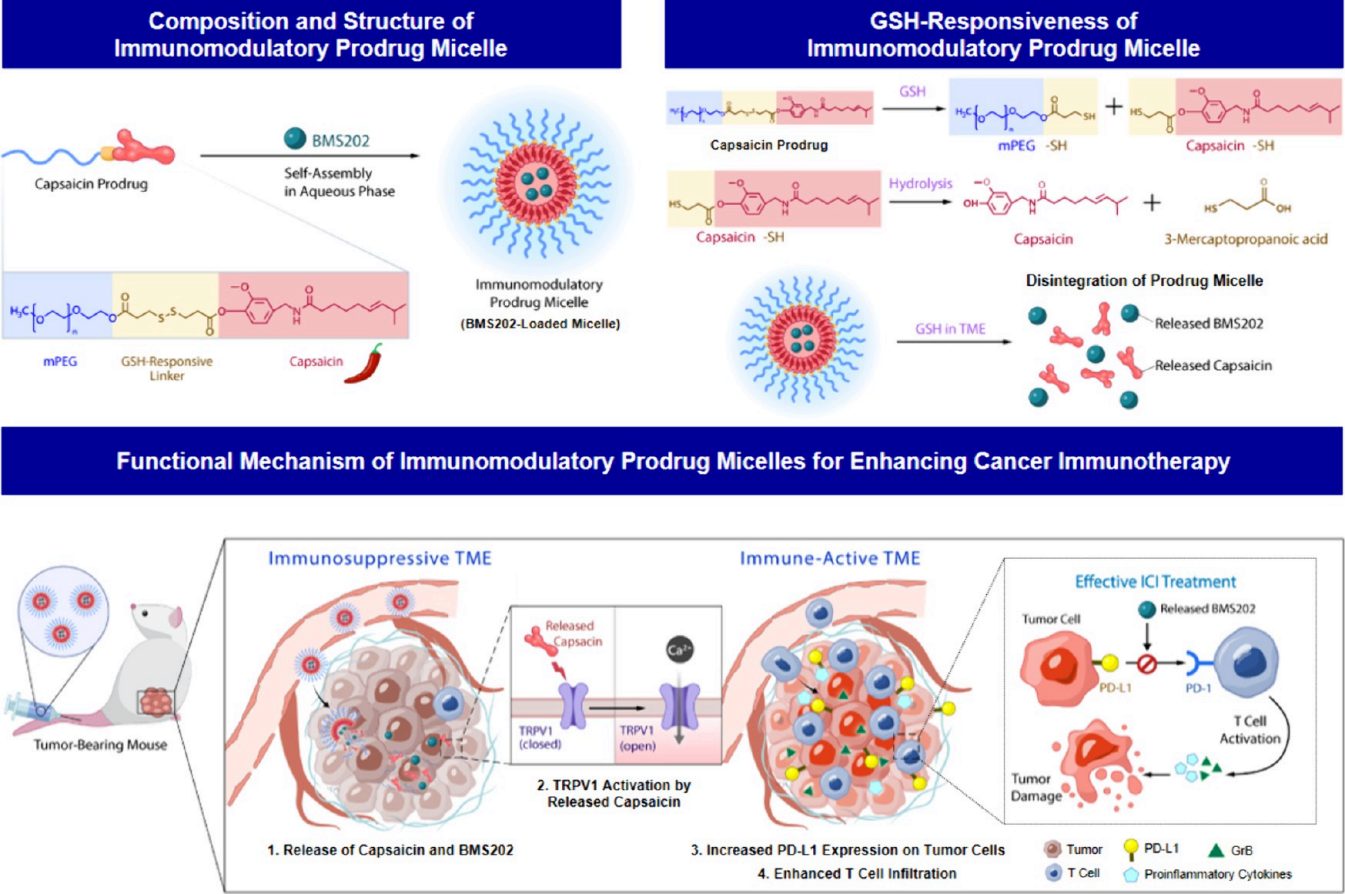
Composition and structure of BMS202-loaded micelle and its responsiveness to GSH with release of capsaicin and BMS202. Illustration of functional mechanism driven by released capsaicin, which transforms the immunosuppressive TME into an immune-active milieu, consequently enhancing the efficacy of ICI treatment in tumor-bearing mice.

Following intravenous administration of the BMS202-loaded micelles to the tumor-bearing mice, they accumulate at the tumor site due to the enhanced permeability and retention (EPR) effect.^28^ Within the GSH-rich TME, a distinctive trait of tumors,^29^ the BMS202-loaded micelles respond by cleaving GSH-responsive disulfide bonds, triggering capsaicin release. This response leads to micelle disintegration and subsequent BMS202 release. The released capsaicin then *in situ* activates TRPV1 in the TME, upregulating the expression of PD-L1 on the tumor cells and increasing the recruitment of the T cells into the TME. Simultaneously, the liberated BMS202 impedes the interaction between PD-L1 on the tumor cells and PD-1 on the T cells, facilitating the activation of the recruited T cells and greatly boosting their immune responses against tumor cells (Figure 1). Through this comprehensive approach, the proposed BMS202-loaded micelles, which mimic the immunomodulatory effects generated by mild heat, introduce an alternative strategy to fine-tune the TME and intensify the effectiveness of cancer immunotherapy.

## Results and Discussion

### Synthesis and Characterization of Capsaicin Prodrug

**Figure 2:**
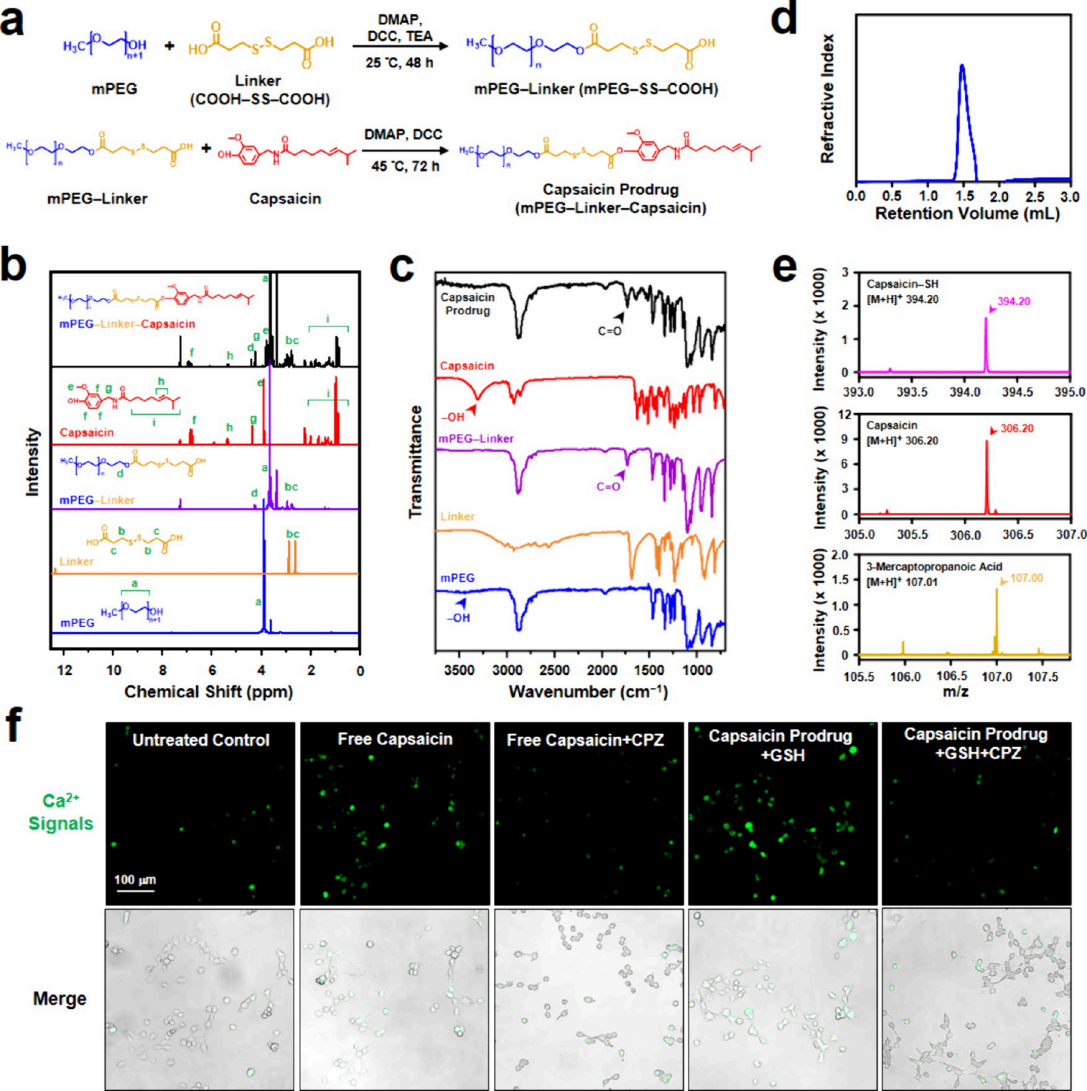
Characteristics of capsaicin prodrug. (a) Synthesis of capsaicin prodrug. (b) ^1^H NMR spectra and (c) FT–IR spectra of mPEG, linker, mPEG–linker, capsaicin, and capsaicin prodrug. (d) GPC trace of capsaicin prodrug. (e) HR–MS analysis of released components from capsaicin prodrug after exposure to GSH. (f) Fluorescence images of Ca^2+^ influx in 4T1 cells following various treatments. DMAP: 4-dimethylaminopyridine; DCC: N, N′-dicyclohexylcarbodiimide; TEA: trimethylamine.

To synthesize the GSH-responsive capsaicin prodrug, a disulfide-containing linker (DTPA or COOH–SS–COOH) was utilized to establish a connection between the polymer (mPEG) and the TRPV1 chemical activator (capsaicin) through a two-step esterification procedure.^30^ As presented in Figure 2a, the esterification reaction occurred between the hydroxyl group (−OH) of mPEG and the carboxyl group (−COOH) of the linker, resulting in the anchoring of mPEG onto the linker (mPEG–linker or mPEG–SS–COOH). To prevent the formation of mPEG–SS–mPEG and enable further conjugation with capsaicin, an excess amount of DTPA was used in comparison to mPEG, aligning with published reference.^30,31^ Consequently, the carboxyl group in the resulting mPEG–linker complex underwent another esterification process with the hydroxyl group of capsaicin, ultimately yielding the as-designed amphiphilic capsaicin prodrug (mPEG–linker–capsaicin).

The chemical structure of the synthesized capsaicin prodrug was characterized by proton nuclear magnetic resonance (^1^H NMR) spectroscopy and Fourier-transform infrared (FT–IR) spectroscopy. In the obtained ^1^H NMR spectra, the proton signal of the carboxylic acid in mPEG–SS–COOH was observed at about 12 ppm (Figure S1), confirming the successful synthesis of mPEG–linker complex.^32^ In addition, the emergence of ester bonds in both the mPEG–linker complex and the capsaicin prodrug (mPEG–linker–capsaicin) was verified by distinct proton signal peaks at 4.25 ppm (peak d). Notably, recognizable signals corresponding to mPEG (peak a), the linker (peaks b and c), and capsaicin (peaks e–i) were also observed in the as-prepared capsaicin prodrug (Figure 2b).

On the other hand, FT–IR spectra showed the absence of the characteristic peak of −OH (3350–3650 cm^–1^) in both the mPEG–linker complex and the capsaicin prodrug. Instead, a new signature peak at 1730 cm^–1^, indicative of the carbonyl group (C=O), appeared in both the mPEG–linker complex and the capsaicin prodrug (Figure 2c). This observation can be attributed to the esterification reaction. The above findings collectively underscore the successful synthesis of the GSH-responsive capsaicin prodrug. Furthermore, the molecular weight of the capsaicin prodrug was assessed via gel permeation chromatography (GPC), resulting in a measurement of 2402 Da, aligning closely with our simulation result of approximately 2435 Da. The presence of a narrow and unimodal GPC trace in Figure 2d further confirms the successful synthesis of the capsaicin prodrug.

GSH is a natural biological reducing agent that can effectively cleave disulfide bonds.^30^ Within the TME, GSH levels (ranging from 0.5 to 10 mM) surpass those typically observed in normal tissues.^29,33^ Accordingly, the capsaicin prodrug, which incorporates the disulfide bond, is expected to remain stable under normal physiological conditions while becoming cleavable within the GSH-rich TME.

To assess the GSH responsiveness of the synthesized capsaicin prodrug, the components released from the capsaicin prodrug upon exposure to aqueous GSH (10 mM) (capsaicin prodrug+GSH) were detected using high resolution mass spectrometry (HR–MS). As illustrated in Figure 1, the disulfide bond within the capsaicin prodrug can be converted to the thiol form (−SH) through the action of GSH,^34^ yielding mPEG–SH and capsaicin–SH. Subsequently, the ester bond in capsaicin–SH undergoes hydrolysis,^34^ releasing capsaicin and forming 3-mercaptopropanoic acid.

In the obtained HR–MS spectra (Figure 2e), following incubation with GSH, the ion peaks, corresponding to capsaicin–SH (*m*/*z* 394.20), capsaicin (*m*/*z* 306.20), and 3-mercaptopropanoic acid (*m*/*z* 107.00), were detected. These results confirm the GSH responsiveness of the capsaicin prodrug, demonstrating the release of capsaicin from the prodrug upon exposure to GSH.

The effectiveness of the released capsaicin from the capsaicin prodrug in activating TRPV1 on 4T1 cells was subsequently investigated. The 4T1 cells are mouse mammary tumor cells with TRPV1 expressed on their cell membranes,^35^ commonly employed as a representative of cold tumors.^8,36^ Upon activation, TRPV1 functions as a nonselective cation channel, allowing the preferential entry of extracellular Ca^2+^ into the cells and leading to an increase in intracellular calcium concentrations.^35^ Therefore, the elevated intracellular calcium levels effectively signify TRPV1 activation. To visualize the intracellular signals associated with the calcium entry, a fluorescent calcium binding dye (Fluo-8 AM) was used in this study.^37^

While untreated cells displayed minimal fluorescence signal, cells treated with either free capsaicin or capsaicin prodrug+GSH (containing a similar amount of capsaicin as in free capsaicin) exhibited a robust fluorescence intensity (Figure 2f). However, in the presence of capsazepine (CPZ), a selective TRPV1 blocker,^18^ the fluorescence intensity was significantly reduced in cells treated with free capsaicin or the capsaicin prodrug. Collectively, these results suggest that the capsaicin released from the capsaicin prodrug in the presence of GSH is equally effective in activating TRPV1 compared to free capsaicin.

### Preparation and Characterization of BMS202-Loaded Micelles

**Figure 3:**
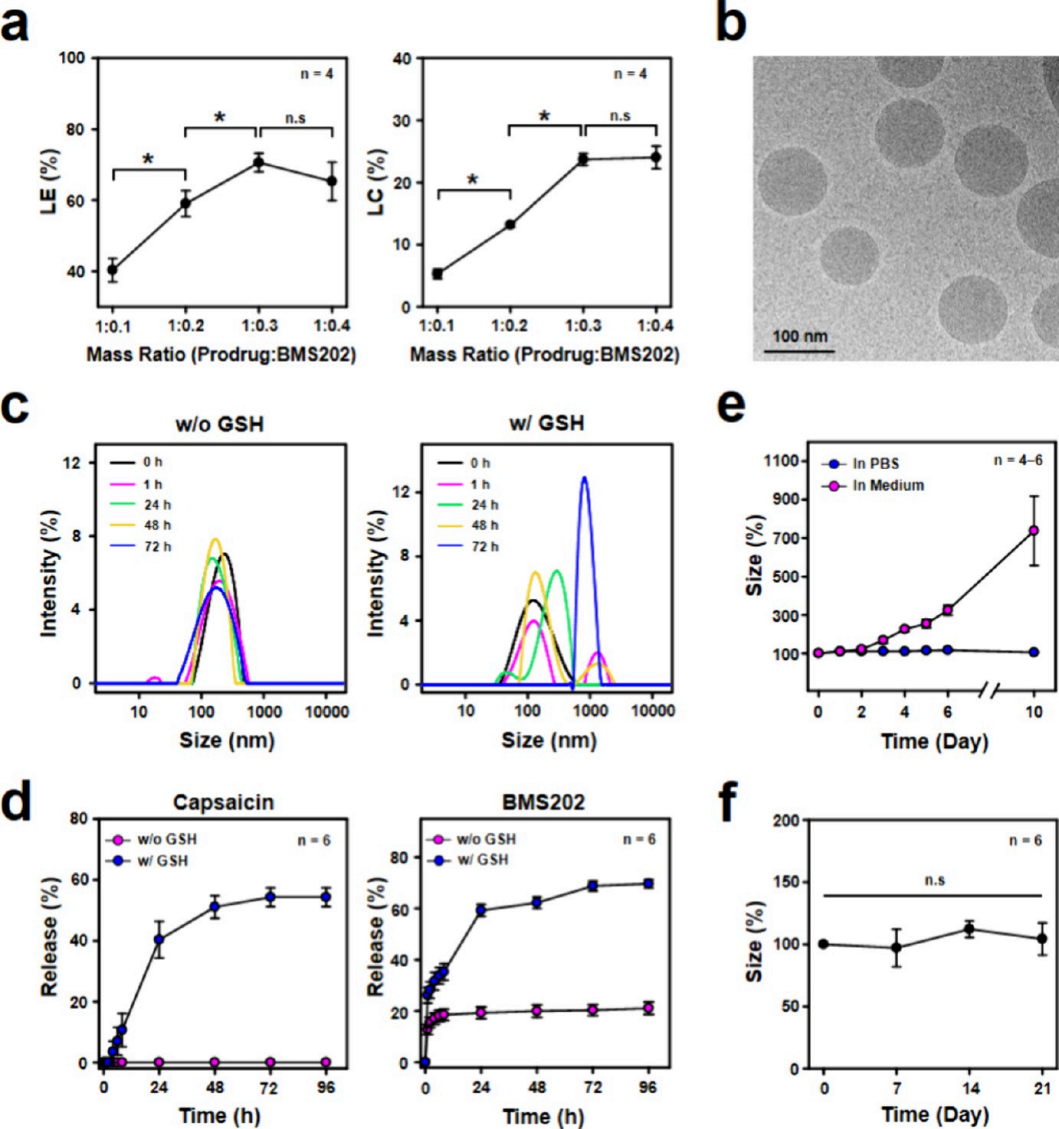
Characteristics of BMS202-loaded micelles. (a) LE and LC of BMS202 in BMS202-loaded micelles prepared with various mass ratios of capsaicin prodrug to BMS202. (b) Cryo-EM image of BMS202-loaded micelles. (c) Size distribution of BMS202-loaded micelles in absence or presence of GSH. (d) Release profiles of capsaicin and BMS202 from BMS202-loaded micelles in absence or presence of GSH at 37 °C. (e) Size changes of BMS202-loaded micelles when incubation with PBS or medium. (f) Size changes of BMS202-loaded micelles when stored in dry powder form at −20 °C for up to 21 days. *: statistically significant (*P* < 0.05). n.s.: not significant (*P* > 0.05).

The BMS202-loaded micelles were prepared by a self-assembly process involving the amphiphilic capsaicin prodrug and the hydrophobic BMS202 with the assistance of sonication. The refinement of the micelle formulation was achieved by carefully adjusting the feeding mass ratio of the capsaicin prodrug to BMS202 during assembly. As the feeding ratio of BMS202 increased, both the loading efficiency (LE) and the loading content (LC) of BMS202 within the micellar structure increased, reaching their peaks at a capsaicin prodrug to BMS202 mass ratio of 1:0.3 (Figure 3a). The formulation optimized at this mass ratio was thus chosen for subsequent investigations. The contents of capsaicin and BMS202 of the optimized BMS202-loaded micelles were 32.3 ± 2.7 and 217.1 ± 16.6 μg/mg micelles (*n* = 5 batches), respectively. The micelles had a spherical morphology, as observed by cryogenic electron microscopy (Cryo-EM, Figure 3b), with a particle size of 147.7 ± 7.7 nm and a zeta potential of 0.7  ±  0.6 mV (*n* = 6 batches) as determined by dynamic light scattering (DLS).

To investigate the GSH responsiveness of the BMS202-loaded micelles, the change in their size distribution in phosphate-buffered saline (PBS, pH 7.4) in the absence or presence of GSH was examined over time by DLS measurements. Figure 3c shows that no significant alterations in size distribution of the BMS202-loaded micelles occurred over a span of 72 h when GSH was absent. Conversely, in the presence of GSH, the size distribution of the micelles became broader over time due to the emergence of larger aggregates. This change could be attributed to the breakage of disulfide linkages between hydrophilic mPEG and hydrophobic capsaicin within the BMS202-loaded micelles, causing the hydrophobic components (capsaicin and BMS202) to aggregate and then precipitate in the aqueous environment.^38^ These findings suggest that the BMS202-loaded micelles maintain considerable stability in the absence of GSH but display disintegration tendencies within an environment enriched with GSH, highlighting their responsiveness to changes in GSH concentration.

The potential impact of GSH-induced disintegration of the BMS202-loaded micelles on the release of capsaicin and BMS202 was further explored. In the absence of GSH, the release of capsaicin demonstrated limited progress, with less than 20% of BMS202 released from the micelles over 96 h. In contrast, upon exposure to GSH, both capsaicin and BMS202 exhibited significantly accelerated release rates, reaching their maximum (ca. 60% for capsaicin and 70% for BMS202) around 72 h (Figure 3d). These experimental results imply a restricted release of capsaicin and BMS202 prior to the micelles encountering the GSH-rich TME, thereby minimizing potential systemic toxicity. Nonetheless, once the micelles reach the GSH-rich TME, an efficient release of capsaicin and BMS202 takes place.

The stability of the prepared BMS202-loaded micelles was assessed by incubating them in both PBS and cell culture medium (RPMI 1640). In PBS, the micelles maintained a consistent particle size over time (Figure 3e). However, when immersed in culture medium, their sizes began to increase after 4 days, likely due to the presence of cysteine in the medium. Cysteine is known to cleave disulfide bonds,^39^ leading to the breakdown of BMS202-loaded micelles and subsequent aggregation. Moreover, the storage capacity of the micelles was evaluated by freeze-drying them into a dry powder state and subsequently storing them at −20 °C. According to Figure 3f, when resuspended in PBS, the particle sizes of the stored micelles displayed no significant alterations over a period of 21 days, suggesting the stability of dry BMS202-loaded micelles throughout the storage duration.

### *In Vitro* Immunomodulatory Effects of BMS202-Loaded Micelles

Enhancing the expression of PD-L1 on tumor cells through physical stimulation with mild heat treatment (43–45 °C) has emerged as a strategic immunomodulatory approach to convert cold tumors into hot tumors.^8^ This process potentially involves the activation of TRPV1 in driving this change. In light of this, an evaluation was conducted to assess whether the chemical stimulation of TRPV1 with capsaicin, released from the BMS202-loaded micelles, could replicate the effects observed with mild heat treatment by boosting PD-L1 expression on tumor cells. Hence, mild heat treatment serves as a benchmark for comparison in this investigation.

Before investigating their immunomodulatory capacity, a comprehensive examination of the cytotoxicity of the BMS202-loaded micelles, their associated components (free capsaicin, free BMS202, and the blank micelles), along with mild heat treatment, was evaluated in 4T1 cells. The blank micelles (Figure S2) are prepared in a similar manner to the BMS202-loaded micelles, but without encapsulating BMS202.

**Figure 4:**
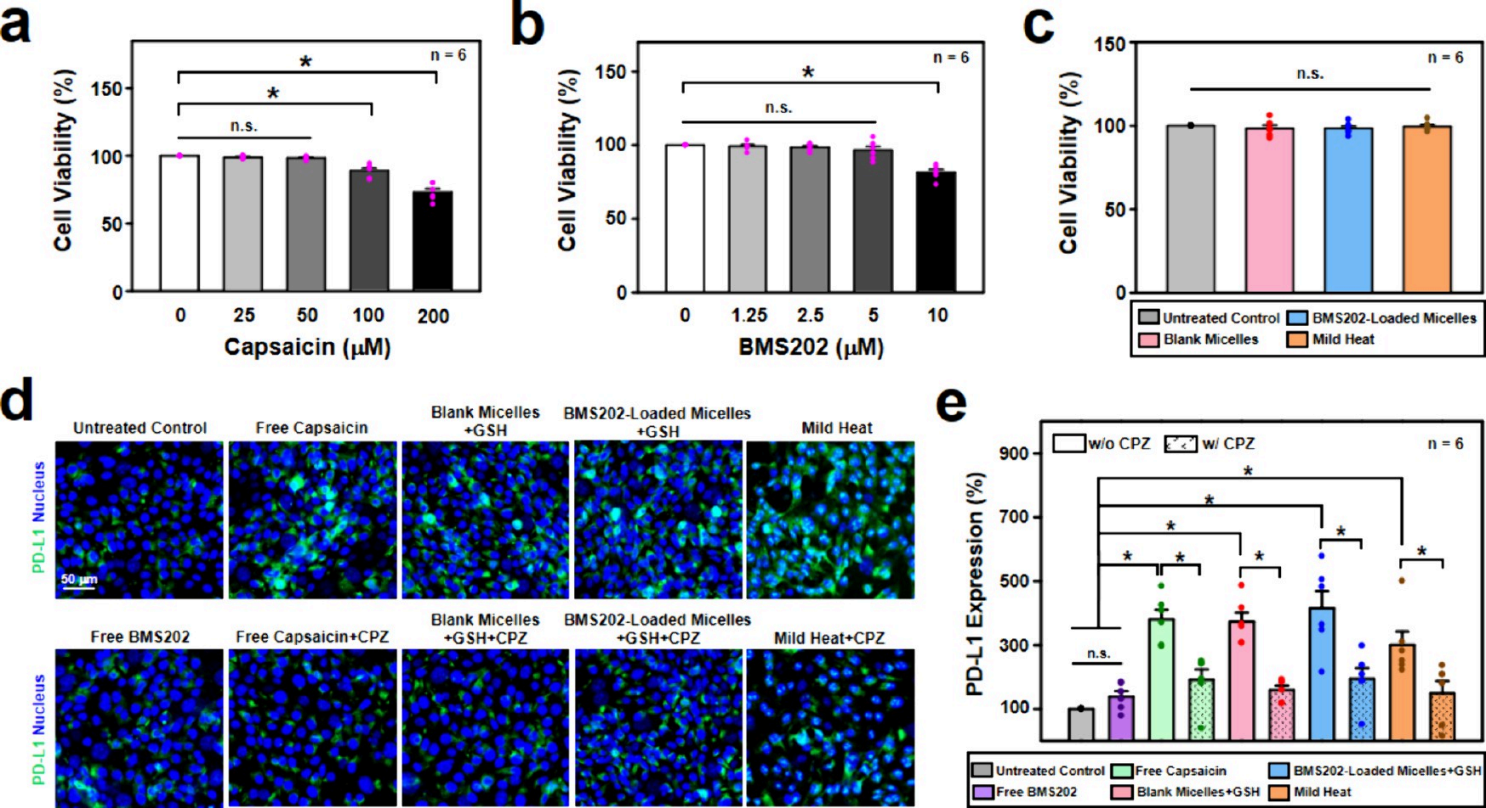
*In vitro* immunomodulation effects. Cell viability of 4T1 cells incubated with (a) free capsaicin and (b) free BMS202 at various concentrations. (c) Cell viability of 4T1 cells following various treatments. (d) Fluorescence images and (e) flow cytometric analysis of cellular levels of PD-L1 expression on 4T1 cells following various treatments. *: statistically significant (*P* < 0.05). n.s.: not significant (*P* > 0.05).

It is known that 10 μM of capsaicin is required to activate TRPV1 in most cancer cell lines.^40^Figure 4a shows that, in comparison to the untreated control group, exposing cells to 25–50 μM of free capsaicin had negligible impact on cell viability (*P* > 0.05). However, treatment with higher concentrations of free capsaicin (50–200 μM) led to a significant reduction in cell viability (*P* < 0.05), in line with previous findings that highlight the cytotoxic effects of high doses of capsaicin.^20,40^ Additionally, Figure 4b indicates that cell viability remained largely unaffected when coincubated with free BMS202 up to a concentration of 5 μM (*P* > 0.05).

To explore their impact on modulating the TME without directly harming tumor cells, the BMS202-loaded micelles containing 50 μM of capsaicin and 2.5 μM of BMS202 were thus selected for subsequent *in vitro* cell studies. Figure 4c demonstrates that both the blank micelles (containing 50 μM of capsaicin) and the BMS202-loaded micelles (containing 50 μM of capsaicin and 2.5 μM of BMS202) showed no noticeable cytotoxicity (*P* > 0.05). Moreover, subjecting the cells to a mild heat treatment did not induce cell death (*P* > 0.05). These findings collectively affirm the nontoxic nature of all components within the BMS202-loaded micelles and validate the safety of employing mild heat treatment.

The investigation then shifted to assess the potential of the BMS202-loaded micelles in enhancing the expression of PD-L1 on 4T1 tumor cells. Immunoblotting results initially confirmed that cells treated with free capsaicin or subjected to mild heat increased PD-L1 expression (Figure S3). Furthermore, the findings from immunocytochemistry and flow cytometry indicated that the untreated control cells and those treated with free BMS202 seldom displayed PD-L1 signals (Figure 4d,e). Conversely, cells incubated with free capsaicin, the blank micelles+GSH, the BMS202-loaded micelles+GSH, or subjected to mild heat showed more prominent PD-L1 signals compared to the untreated control and free BMS202 treatment (Figure 4d,e, *P* < 0.05). However, these signals markedly diminished when cells were pretreated with CPZ (*P* < 0.05). Notably, both the blank micelles and the BMS202-loaded micelles exhibit immunomodulatory effects, possibly attributed to the release of capsaicin from the micelle structure in the presence of GSH (Figure 3d).

The *in vitro* data suggest that chemical stimulation with free capsaicin or capsaicin released from the blank micelles or the BMS202-loaded micelles, rather than free BMS202, can replicate the effects generated from the physical stimulation with mild heat treatment. This replication resulted in the upregulation of PD-L1 expression on tumor cells through the activation of TRPV1. It is noted that the similar phenomenon could be also observed in bladder cell lines.^41^ These promising findings suggest the considerable potential of the BMS202-loaded micelles in eliciting *in vivo* antitumor responses.

### *In Vivo* Antitumor Efficacy of BMS202-Loaded Micelles

Before verifying the *in vivo* antitumor efficacy of the BMS202-loaded micelles, an initial investigation was conducted to study their pharmacokinetics and biodistribution following tail vein administration. The pharmacokinetics of the prodrug micelles in tumor-free mice were examined using a hydrophobic fluorescent dye (coumarin 6, C6), which served as a model drug for BMS202. It was encapsulated within the prodrug micelles (C6-loaded micelles) through a process similar to the one employed for the BMS202-loaded micelles. Free C6 was utilized as a control. As presented in Figure S4, free C6 was rapidly eliminated from the blood after intravenous injection. On the contrary, the circulation of C6 was prolonged when loaded into micelles, suggesting that a drug (e.g., BMS202) carried in the prodrug micelles remains stable during transportation to the tumor site.

The biodistribution of the prodrug micelles was then conducted in a tumor-bearing mouse model. The experimental mice were subcutaneously inoculated with 4T1 cells into their right flank to induce a primary tumor. To visualize *in vivo* biodistribution of the prodrug micelles, IR-780 was used as a tracer and encapsulated within the prodrug micelles to form IR-780-loaded micelles. An *in vivo* imaging system (IVIS) was applied to capture images of the entire animal at predetermined time points, as well as images of *ex vivo* major organs harvested at 24 h postinjection.

**Figure 5:**
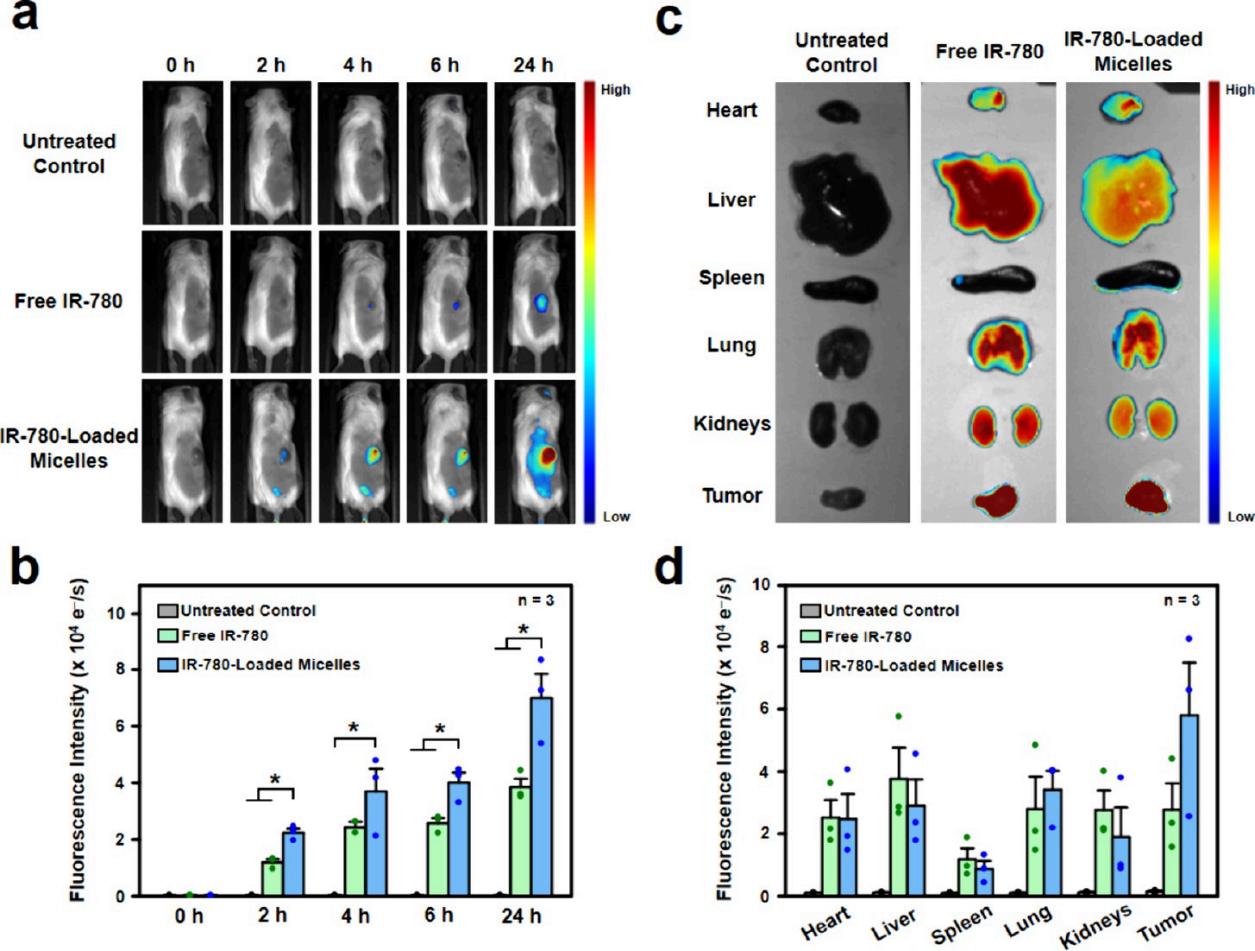
*In vivo* biodistribution of IR-780-loaded micelles in tumor-bearing mice. (a) IVIS images and (b) corresponding quantified results of fluorescence signals from IR-780 at different time points after intravenous injection of free IR-780 or IR-780-loaded micelles. (c) *Ex vivo* IVIS images and (d) corresponding quantified results of IR-780 from major organs and tumors 24 h after injection. *: statistically significant (*P* < 0.05).

In the IVIS images of entire animal, it was evident that mice intravenously injected with the IR-780-loaded micelles emitted a significantly stronger accumulated fluorescence signal at tumor sites over time when compared to both the untreated mice and the mice administered with free IR-780 (Figure 5a,b, *P* < 0.05). In the *ex vivo* analysis, a higher fluorescence signal was detected in the tumor from the test mouse treated with the IR-780-loaded micelles, in comparison to major organs (Figure 5c,d), which aligns with the results obtained from the entire animal.

These observations emphasize the increased tumor accumulation of the IR-780-loaded micelles, which is likely due to the EPR effect. Typically, nanoparticles with sizes smaller than 500 nm can take advantage of the enhanced permeability of vasculature in solid tumors, enabling them to passively diffuse through the vascular gaps and consequently accumulate within the TME.^42^ The accumulation of the prodrug micelles within the tumor could realize the controlled release of drug in response to the GSH-rich TME (Figure 3d). This process aims to elevate drug concentration at the site of action while minimizing systemic exposure in healthy tissues, potentially reducing the adverse effects.

Physical stimulation with mild heat has been acknowledged for its ability to reshape the TME and enhance the effectiveness of ICI therapy against tumors.^8,11,12^ To explore whether chemical stimulation employing capsaicin released from the BMS202-loaded micelles can imitate these effects of mild heat, this study investigated the therapeutic impact of the BMS202-loaded micelles on primary tumors. Tumor-bearing mice were randomly divided into six treatment groups: untreated control, free capsaicin, free BMS202, a combination of free capsaicin and free BMS202 (capsaicin+BMS202), blank micelles, and BMS202-loaded micelles. Each treatment was administered intravenously into the tail vein once every 3 days, specifically on days 0, 3, 6, 9, 12, 15, and 18, over a 20-day period. This administration frequency was chosen based on the release profiles of capsaicin and BMS202 from the BMS202-loaded micelles following exposure to GSH, with both compounds reaching their maximum release approximately within 3 days (Figure 3d). Throughout the treatment period, the tumor volume of each test mouse was monitored every other day.

**Figure 6:**
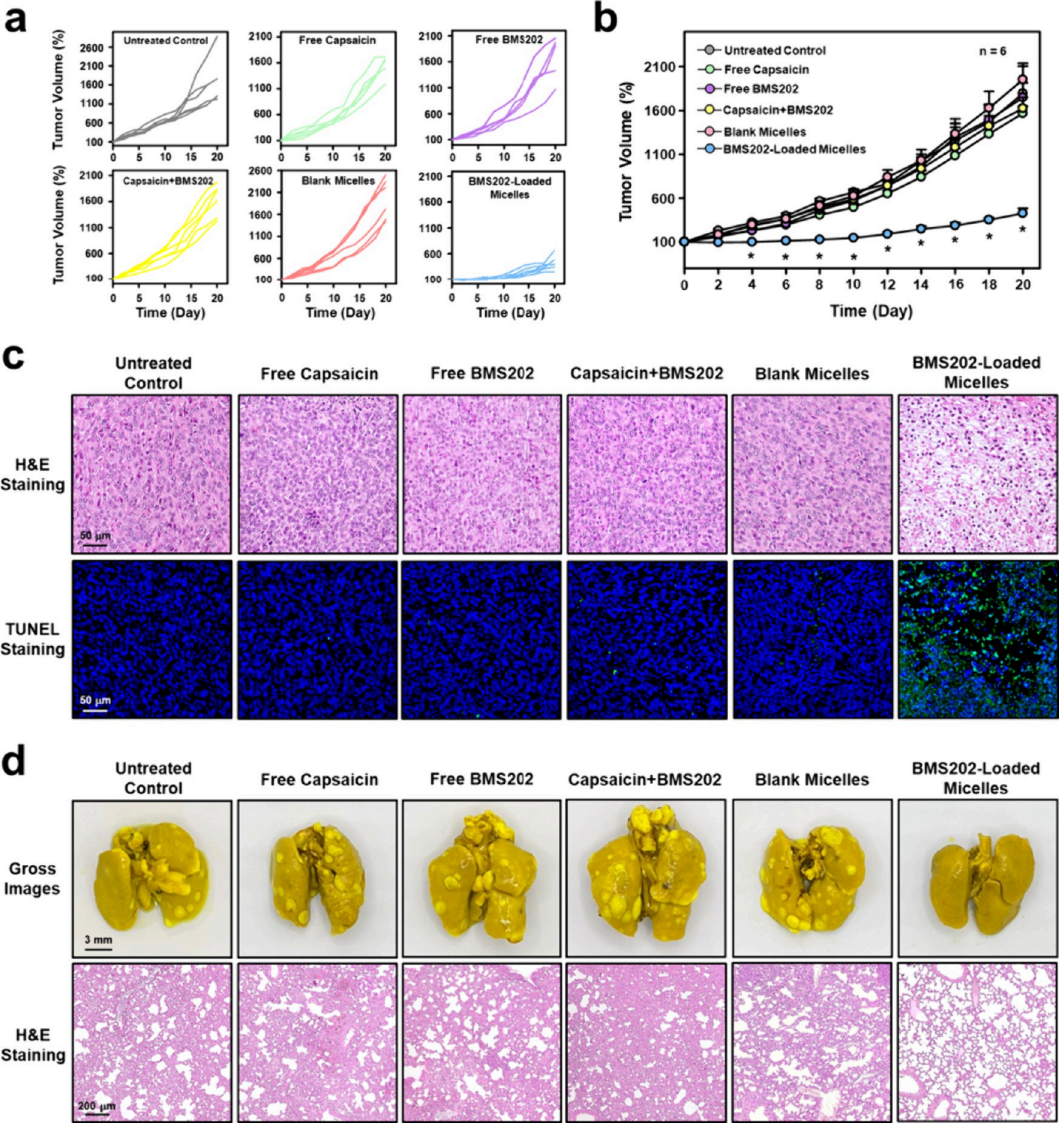
*In vivo* antitumor efficacy. (a) Individual and (b) average tumor growth curves of primary tumor of test mice undergoing various treatments. (c) H&E and TUNEL staining of primary tumor tissues following various treatments. (d) Gross appearances and H&E staining of lung tissues in tumor-bearing mice that have been administered various treatments.

As presented in Figure 6a,b, the tumor volumes of mice receiving free capsaicin, free BMS202, capsaicin+BMS202, or blank micelles did not exhibit significant differences compared to that of the untreated control group during the treatment period. In contrast, treatment with the BMS202-loaded micelles substantially reduced tumor volumes at various monitoring points (days 4, 6, 8, 10, 12, 14, 16, 18, and 20, Figure 6b, *P* < 0.05). At the end of the observation period, results obtained from hematoxylin and eosin (H&E) and TUNEL staining of tumor slices also exclusively revealed severe cell death in the group that received the BMS202-loaded micelles (Figure 6c). These results indicate that only the BMS202-loaded micelles can effectively inhibit the growth of primary tumors.

Research studies have shown that 4T1 breast cancer cells demonstrate a pronounced aggressiveness in their growth pattern. This aggressive behavior enables a rapid metastasis from the primary tumor site to various organs, with a notable preference for the lungs. This, in turn, triggers the spontaneous initiation of metastatic tumor growth within the lung area.^43,44^ As shown in Figure 6d, at the experimental end point, lung metastatic nodules were observed in all test groups, except in the group treated with the BMS202-loaded micelles, revealing the suppressive effect of these micelles on the development of lung metastasis. Together, the BMS202-loaded micelles appear to be effective in inhibiting the growth of both primary and metastatic tumors, aligning well with the efficacy observed in the combined effects of mild heat and ICI treatment that previously published.^8,11,12^

### *In Vivo* Immunomodulatory Effects of BMS202-Loaded Micelles

**Figure 7:**
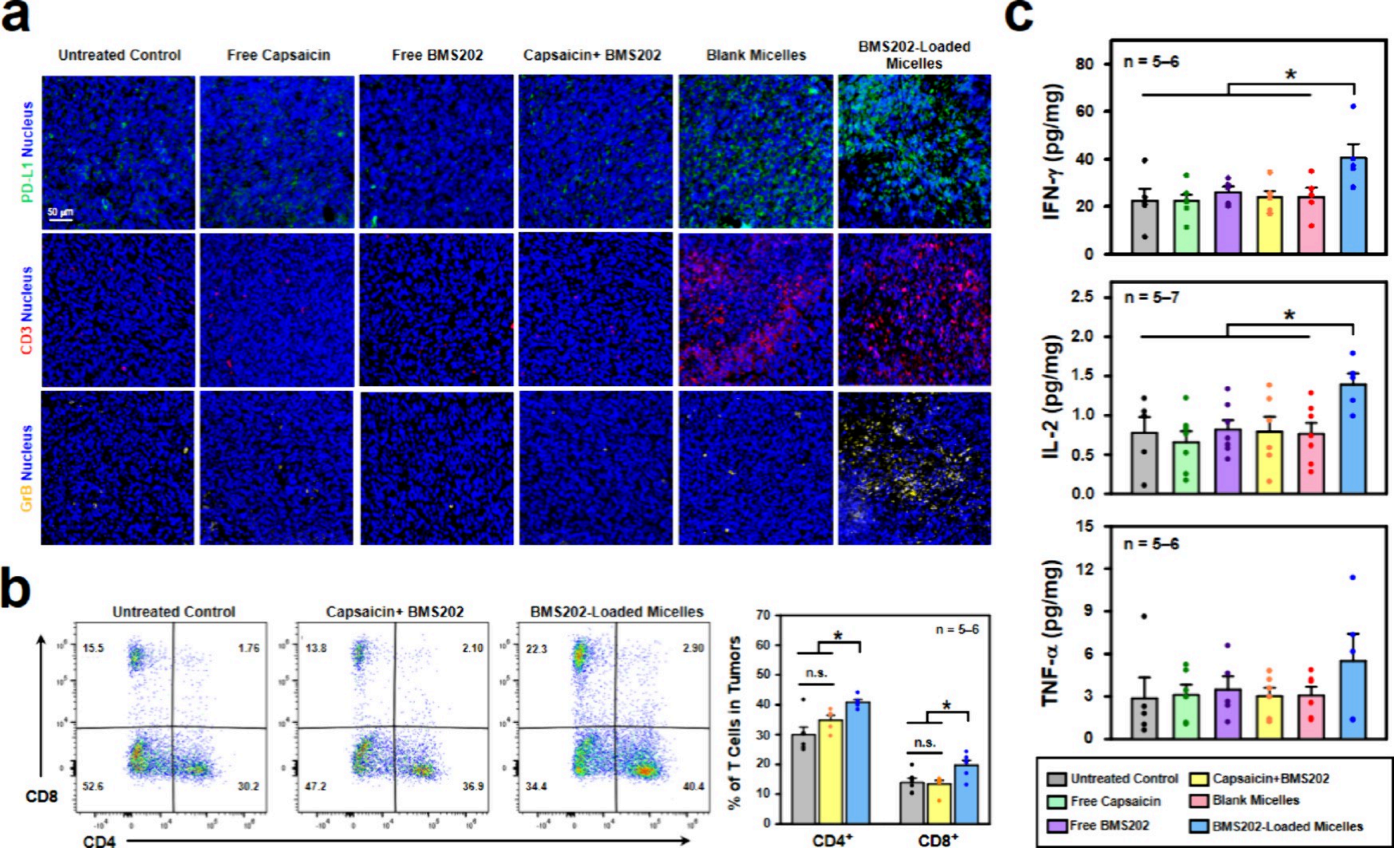
*In vivo* immunomodulation effects. (a) Fluorescence images of primary tumor sections stained for PD-L1, CD3 or GrB. (b) Flow histogram plots and corresponding quantified results showing percentages of CD4^+^ and CD8^+^ T cells in TME (gated on CD3^+^ T cells) following various treatments. (c) Inflammatory cytokine levels of IFN-γ, IL-2, and TNF-α in primary tumors retrieved from test mice.

Mild heat has been observed to modulate the TME from an immunosuppressive state to an immune-active one. This modulation involves enhancing the expression of PD-L1 on tumor cells and promoting the recruitment of T cells into the TME.^8^ Specifically, the maturation of dendritic cells (DCs) plays a role in enhancing T cell infiltration.^45^ To investigate the immunomodulatory potential of the BMS202-loaded micelles for reshaping the TME, tumor slices from primary tumors collected across various treatment groups underwent immunofluorescence staining for PD-L1, CD3 (a T cell marker)^13^ or CD86 (a mature DC marker).^45^ The analysis of the findings reveal that tumor treated with free capsaicin, free BMS202, or capsaicin+BMS202 did not show differences in the fluorescence intensity of PD-L1, CD3 and CD86 when compared to those from the untreated control group (Figures 7a and S5). This suggests that treatment with free capsaicin, free BMS202, or capsaicin+BMS202 fails to alter the immunosuppressive TME. As a result, these treatments are unable to inhibit primary and metastatic tumor growth (Figure 6a–d).

Conversely, tumors treated with either the blank micelles or the BMS202-loaded micelles exhibited higher PD-L1 expression, increased CD3^+^ T cell, and mature DC infiltration in comparison to the other control groups (Figures 7a and S5). This indicates that both types of micelles efficiently transform the immunosuppressive TME (cold tumors) into an immune-active state (hot tumors). This transformation likely occurred due to the localized release of capsaicin from the micelle structure in the GSH-rich TME (Figure 3d). The released capsaicin could induce the activation of TRPV1 in the TME, triggering the process of immunomodulation (Figure 4d,e).

Recognizing the crucial roles of CD4^+^ and CD8^+^ T cells in eliciting immune responses during antitumor therapy,^8,23^ their individual presence in the TME was distinguished and quantified using flow cytometry. As shown in Figure 7b, the group treated with the BMS202-loaded micelles displayed significantly increased populations of both CD4^+^ and CD8^+^ T cells compared to the control treatments (*P* < 0.05). Moreover, the absolute counts of CD4^+^ and CD8^+^ T cells within 1 × 10^6^ CD45^+^ positive cells were much higher in the BMS202-loaded micelle-treated group (Figure S6). This observation strongly suggests that the BMS202-loaded micelles possess the capability to effectively recruit these cytotoxic T cells into the TME. A previous study demonstrates that TRPV1 activation might enhance T cell infiltration.^46^ This implies that the ability of BMS202-loaded micelles to attract T cells into the TME may be attributed to the activation of TRPV1 by the capsaicin released from these micelles.

It is worth noting that ICI is known for its potential in activating T cells.^8,11,12^ The activated T cells in the TME can secrete GrB and proinflammatory cytokines to destroy tumors.^47^ As demonstrated in Figure 7a,c, only the group that received the BMS202-loaded micelles displayed elevated GrB signals and considerably higher levels of proinflammatory cytokines, notably IFN-γ (*P* < 0.05) and IL-2 (*P* < 0.05), compared to those subjected to control treatments. Additionally, while not reaching statistical significance, there was also a discernible trend toward elevated TNF-α levels.

Although tumors treated with the blank micelles (lacking BMS202) showed potential in reshaping the TME by enhancing PD-L1 expression on tumor cells and increasing T cell recruitment (Figure 7a), they failed to produce GrB (Figure 7a) and notable proinflammatory cytokines (Figure 7c). This points to their inability to sufficiently activate T cells due to the absence of BMS202, the ICI used in the study, resulting in their failure to impede tumor growth (Figure 6a–6d). On the contrary, when accompanied by BMS202, the BMS202-loaded micelles effectively engaged and activated T cells, leading to increased secretion of GrB and proinflammatory cytokines, successfully eliminating the tumors.

Studies have highlighted the significance of DC maturation and enhanced T cell infiltration in establishing a long-term antitumor immune memory, a crucial factor for preventing the tumor reoccurrence and metastasis.^48−50^ Notably, with the assistance of BMS202, BMS202-loaded micelles can induce DC maturation (Figure S5), enhance T cell infiltration (Figures 7a,b and S6), and hinder the development of lung metastasis (Figure 6d), suggesting that BMS202-loaded micelles have the potential to generate immunological memory. Taken together, the synergistic impact of capsaicin and BMS202 for antitumor responses observed in the BMS202-loaded micelles-treated group significantly resembles the effects seen with mild heat combined with ICI treatment.

*In vivo* safety of the BMS202-loaded micelles was also carried out in the study. Throughout the treatment periods, there was no notable variance in body weight among the different treatment groups (*P* > 0.05, Figure S7). Moreover, a comprehensive histological examination of the major organs, extracted from various treatment groups at the experimental end point, revealed no evident abnormalities or signs of inflammation (Figure S8). Simultaneously, serum levels of alanine aminotransferase (ALT), aspartate aminotransferase (AST), urea nitrogen (BUN) and creatinine in the mice treated with BMS202-loaded micelles remained within the normal range. These levels were comparable to those observed in untreated control mice (*P* > 0.05, Figure S9). These outcomes underscore the biosafety and biocompatibility of the developed BMS202-loaded micelles.

Applying the physical stimulation of mild heat for raising TME temperature to a range of 43–45 °C shows potential in converting the immunosuppressive TME into an immune-active state.^8^ This process may involve the activation of TRPV1 in the TME. Conventional methods for applying mild heat heavily rely on external heat sources, including near-infrared light coupled with photosensitizers, ultrasonic systems, and microwave.^8,23,51,52^ Nevertheless, these methods encounter limitations, particularly in achieving uniform heating across entire tumor volumes due to their restricted tumor penetration and the requirement for precise knowledge of tumor positioning prior to applying heat sources.^53,54^ Furthermore, maintaining accurate temperature regulation within the narrow range of 43–45 °C poses challenges, heightening the potential risk of harming surrounding healthy tissues, such as inducing skin burns.^53,54^

In response to the aforementioned limitations of physical stimulation with mild heat, this study suggests employing capsaicin for chemical stimulation to activate TRPV1 in the TME. This strategy is accomplished by an intelligent immunomodulatory mechanism facilitated by the BMS202-loaded micelles. These micelles, within the GSH-rich TME, release the TRPV1 chemical activator, capsaicin, replicating the immunomodulatory effects of mild heat. This replication reshapes the TME into an immune-active state, evidenced by the overexpression of PD-L1 on tumor cells (Figure 4d,e, Figures 7a) and increased T cell recruitment into the TME (Figures 7a,b). Simultaneously, the liberated BMS202 assists in activating recruited T cells to release GrB (Figure 7a) and proinflammatory cytokines (Figure 7c) to destroy the tumors (Figure 6a–d). This synergistic effect holds immense potential for enhancing immune responses within the TME, fostering T cell-mediated attacks on tumors.

Importantly, the utilization of BMS202-loaded micelles eliminates the dependence on external heat sources, rendering their application unrestricted by tumor size or position. Furthermore, the GSH-responsiveness of the BMS202-loaded micelles ensures the localized release of capsaicin and BMS202 exclusively within the TME, thereby reducing the risk for adverse side effects (Figures S7–S9).

## Conclusions

This work presents an innovative method involving GSH-responsive immunomodulatory prodrug micelles (BMS202-loaded micelles) delivered to the tumor site. In the GSH-rich TME, the micelles respond and disintegrate, releasing capsaicin and BMS202. The released capsaicin chemically stimulates TRPV1 in the TME, imitating the immunomodulatory effects induced by physical stimulation with mild heat. This process reshapes the TME by enhancing PD-L1 expression on tumor cells and attracting more T cells into the TME. Simultaneously, the liberated BMS202 promotes T cell-mediated antitumor responses. Employing the released capsaicin for chemical stimulation offers localized immune modulation within the TME, overcoming limitations associated with physically applying mild heat, such as uneven heating of tumors, needing precise tumor positioning, and potential harm to healthy tissues. This approach holds significant promise for potentially treating a wide range of immunologically cold tumors by converting them into hot tumors, consequently enhancing cancer immunotherapy across diverse cancer types.

## Experimental Section

### Materials

DTPA, mPEG (Mn 2000), CPZ, GSH, C6, and IR-780 were purchased from Sigma-Aldrich (St. Louis, MO, USA). BMS202 was obtained from MedChemExpress (Monmouth Junction, NJ, USA), while capsaicin was purchased from Tokyo Chemical Industry (Tokyo, Japan). Acetyl chloride, N,N′-dicyclohexylcarbodiimide (DCC), 4-dimethylaminopyridine (DMAP), and triethylamine (TEA) were acquired from Alfa Aesar (Ward Hill, MA, USA). 4T1 cells were obtained from the American Type Culture Collection (ATCC, Manassas, VA, USA). All other chemicals and reagents were of analytical grade.

### Synthesis and Characterization of Capsaicin Prodrug

The GSH-responsive capsaicin prodrug was prepared using a two-step esterification procedure.^29,30^ First, to synthesize the mPEG–linker (Figure 2a), DTPA (5 mmol), DCC (5 mmol), DMAP (0.5 mmol), and TEA (2.5 mmol) were dissolved in anhydrous dichloromethane (DCM, 30 mL) under an inert atmosphere, and stirred on ice for 2 h. Subsequently, a solution of mPEG (1 mmol) in DCM (10 mL) was added and continued stirring for an additional 48 h at 25 °C. Following filtration, the filtrate was concentrated under a reduced pressure by a rotary evaporator (N-1200A, EYELA, Tokyo, Japan) and then precipitated in diethyl ether. The resulting product was purified through a dissolution–precipitation procedure with DCM/diethyl ether twice. Finally, the purified mPEG–linker was dried overnight in a vacuum oven for further uses.

To create the capsaicin prodrug (mPEG–linker–capsaicin, Figure 2a), the purified mPEG–linker (1 mmol), DMAP (0.5 mmol) and capsaicin (1.5 mmol) were dissolved in DCM (40 mL) and stirred on ice for 30 min, followed by the addition of DCC (1 mmol in DCM) for another 72 h of stirring at 45 °C. Thereafter, the mixture was filtered and concentrated under a reduced pressure, which was then purified by dialysis against deionized (DI) water for 3 days. The purified capsaicin prodrug was then acquired through lyophilization.

The chemical structures of the as-synthesized mPEG–linker and capsaicin prodrug were analyzed using ^1^H NMR spectrometry (Bruker AVANCE 600 MHz NMR spectrometer, Bruker BioSpin, Billerica, MA, USA), FT–IR spectrometry (Nicolet iS50 spectrometer, Thermo Fisher Scientific, Waltham, MA, USA). The molecular weight and the polydispersity of the capsaicin prodrug were determined via GPC (Viscotek GPC System, Malvern Panalytical, Malvern, UK). The GSH-responsiveness of the capsaicin prodrug was also evaluated. Briefly, the capsaicin prodrug was incubated with GSH (10 mM) at 37 °C for 3 days. The resulting samples were then collected, lyophilized, and analyzed using HR–MS (TripleTOF 6600, SCIEX, Foster City, CA, USA).

### Preparation and Characterization of BMS202-Loaded Micelles

The BMS202-loaded micelles were prepared by a self-assembly process involving the amphiphilic capsaicin prodrug and BMS202. The capsaicin prodrug (5 mg) and BMS202 (1.5 mg) were added to dimethyl sulfoxide (DMSO, 55.8 μL) and sonicated in an ice bath for 30 min Afterward, the mixture was added dropwise to the DI water, which was vigorously stirred at 4 °C for 1 h. Then, the obtained micelles were purified by dialysis against DI water. The purified BMS202-loaded micelles were lyophilized and stored at −20 °C for further analysis.

The morphology of the BMS202-loaded micelles was observed by cryo-EM (Tecnai G2 F20 TWIN, FEI, Hillsboro, OR, USA), and their zeta potentials and sizes in the absence or presence of GSH (10 mM) were measured using DLS (Litesizer 500, Anton Paar, Graz, Austria).

To examine the LE and LC of capsaicin or BMS202 within the BMS202-loaded micelles, the weighted samples underwent incubation in aqueous GSH (200 mM) at 37 °C for 3 days, aiming to release all the conjugated capsaicin and encapsulated BMS202. The amounts of capsaicin and BMS202 were determined using high-performance liquid chromatography (HPLC, 1260 infinity II, Agilent Technologies, Santa Clara, CA, USA), and subsequently, the LE and LC in the BMS202-loaded micelles were calculated through following equations.
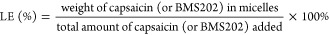

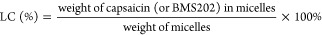
To investigate the release of capsaicin or BMS202 from the BMS202-loaded micelles, the micelle solution was placed in a dialysis bag (MWCO 12000), which was then immersed in PBS in the absence or presence of GSH (10 mM). This system was gently shaken at 37 °C. At predetermined time points, the dialysate was collected and replaced with fresh soaking media. The collected dialysate was further diluted with Tween 20 (0.5%), and the released capsaicin and BMS202 were detected using HPLC.

### Cell Culture

4T1 cells were maintained in RPMI 1640 medium (Corning, Glendale, AZ, USA) supplemented with 10% fetal bovine serum (Corning) and 1% penicillin/streptomycin (Corning) in a humidified 5% CO_2_ atmosphere at 37 °C.

### Calcium Imaging

4T1 cells were initially seeded at a density of 4 × 10^4^ cells/well in eight-well chamber slides (ibidi, Gräfelfing, Germany). After a 24-h incubation period, the cells were subjected to various treatment conditions. These treatments included media containing different components, including free capsaicin (50 μM in 0.025% DMSO), free capsaicin+CPZ (15 μM), capsaicin prodrug (containing 50 μM of capsaicin)+GSH (10 mM) or capsaicin prodrug+GSH+CPZ, for a duration of 2 h. Following the respective treatment periods, the cells were carefully washed three times with PBS. Subsequently, they were stained with a Ca^2+^ indicator, Fluo-8 (5 μM, Abcam, Cambridge, MA, USA), for 30 min at 37 °C. After another three washes with PBS, the fluorescence signals were observed using a fluorescence microscope (Axio Observer 7, Carl Zeiss, Jena, Germany)

### Evaluation of Cytotoxicity

To evaluate the cytotoxicity of capsaicin, BMS202, blank micelles, BMS202-loaded micelles, and mild heat treatment, 4T1 cells were seeded in 96-well plates (5 × 10^4^ cells/well). Twenty-four hours later, the cells were exposed to varying concentrations of capsaicin (0–200 μM), BMS202 (0–10 μM), blank micelles (containing 50 μM of capsaicin), or BMS202-loaded micelles (containing 50 μM of capsaicin and 2.5 μM of BMS202) for another 24 h. Additionally, some cells were subjected to mild heat (43–45 °C) for 10 min. Following the treatments, cell viability was assessed using a CCK-8 assay kit (Dojindo, Kumamoto, Japan).

### *In Vitro* Immunomodulatory Effects

The *in vitro* immunomodulatory effects of the BMS202-loaded micelles were examined by investigating the expression of PD-L1 on tumor cells using immunoblotting, immunofluorescence staining and flow cytometry. For immunoblottng, cells incubated with various treatments for 24 h were lysed by RIPA lysis buffer (Merck-Millipore, Rahway, NJ, USA) containing a protease inhibitor cocktail (Merck-Millipore). Subsequently, proteins in the cell lysates were electrophoresed on a 4–12% Bis-Tris gel (SMOBIO, Hsinchu, Taiwan), transferred onto a polyvinylidene difluoride membrane (Bio-Rad Laboratories, Hercules, CA, USA), and blocked with bovine serum albumin (BSA, 2.5%, APOLO Biochem, Rochester NY, USA) at room temperature for 1 h. The membrane was then incubated with an anti-PD-L1 antibody (ab213480, 1:1000, Abcam) at 4 °C overnight, followed by incubation with a secondary antibody (HRP-conjugated antirabbit IgG, 1:10000, Biolegend, San Diego, CA, USA). The expression of PD-L1 was detected by chemiluminescence substrates (Bio-Rad Laboratories), and the chemiluminescence signal was observed using a biospectrum imaging system (UVP, Upland, CA, USA).

For immunofluorescence staining, cells that had undergone various treatments for 24 h were subjected to immunofluorescent staining with anti-PD-L1 antibody (ab213480, 1:250, Abcam, Cambridge, MA, USA) and further stained with secondary antibody (Alexa Flour 488-conjugated goat antirabbit IgG, 1:1000, abcam). The levels of expressed PD-L1 were visualized by a fluorescence microscope (Axio Observer 7).

In the case of flow cytometry, cells after treatments were harvested, fixed with 4% paraformaldehyde for 20 min, and blocked by anti-CD16/32 antibody (101301, 1:25, BioLegend, San Diego, CA, USA) for 15 min. Afterward, cells were stained with PE-conjugated anti-PD-L1 antibody (155404, 1:25, BioLegend), and the levels of PD-L1 expression were measured using flow cytometry (FACSVerse Cell Analyzer, BD Biosciences, San Jose, CA, USA).

### Animal Study

Female BALB/c mice, aged 6–8 weeks and weighing 18–20 g, were procured from the National Laboratory Animal Center (Taipei, Taiwan). The animal experiments adhered to the guidelines outlined in the “Guide for the Care and Use of Laboratory Animals” provided by the Institute of Laboratory Animal Resources, National Research Council, published by the National Academy Press in 2011. The Institutional Animal Care and Use Committee of Academia Sinica granted approval for all animal study protocols. To establish a tumor-bearing model, 4T1 cells (2 × 10^6^ cells in 100 μL of PBS) were subcutaneously inoculated into the right flank of the experimental mice. Seven days later, when the tumor volumes reached 50 mm^3^, the mice were randomly assigned into various treatment groups.

### Pharmacokinetics of C6-Loaded Micelles

In the pharmacokinetics study, tumor-free mice received intravenous injections via the tail vein with free C6 or the C6-loaded micelles (containing an equal dose to free C6). Blood samples were taken from the submandibular vein at predetermined time points (1 min, 15 min, 30 min, 1, 2, 4, 6, and 24 h) after injection and placed in EDTA vacutainers (BD Biosciences). The fluorescence intensity of C6 was subsequently measured using a spectrophotometer (Synergy HTX, BioTek, Santa Clara, CA, USA).

### Biodistribution of IR-780-Loaded Micelles

In the biodistribution study, tumor-bearing mice were intravenously injected with free IR-780 or the IR-780-loaded micelles (containing an equal dose to free IR-780). The gradual accumulation of IR-780 in the tumor over specific time intervals (0, 2, 4, 6, and 24 h) was visualized by IVIS (MILabs, Houten, Netherlands). Twenty-four hours after injection, the mice were sacrificed, and the major organs and tumors were harvested to obtain IR-780 fluorescence signals using IVIS.

### *In Vivo* Antitumor Efficacy

To evaluate the antitumor efficacy of each treatment, tumor-bearing mice were randomly divided into the following groups: untreated control, free capsaicin (0.35 mg/kg), free BMS202 (2.5 mg/kg), capsaicin+BMS202, blank micelles (containing an equal dose to free capsaicin), and BMS202-loaded micelles (containing an equal dose to capsaicin+BMS202). Each treatment was given via intravenous injection into the tail vein every 3 days over a 20-day period, beginning on days 0, 3, 6, 9, 12, 15, and 18. During the treatment period, the body weight and tumor volume of each test mouse were monitored every other day. The tumor volume was estimated as width^2^ × length × 0.5.^22^

At the experimental end (day 20), the primary tumors as well as major organs, including heart, lungs, liver, spleen, and kidneys, were retrieved. These tissues, with the exception of the lungs, were then preserved by fixation in 4% paraformaldehyde, followed by embedding in paraffin and sectioning for H&E staining. Regarding the lungs, upon retrieval, they were submerged in Bouin’s solution (Sigma) for 6 h, and photographs were taken to facilitate the macroscopic examination for metastatic nodules. Subsequently, the lung tissues underwent paraffin embedding and sectioning for H&E staining.

### Immunofluorescence Staining of PD-L1, CD3, and GrB

To assess *in vivo* immunomodulatory effects, tumor sections from primary tumors gathered across various treatment groups were subjected to immunofluorescence staining against PD-L1, CD3 or GrB. In brief, tumor sections were first deparaffinized and then subjected to heat-mediated antigen retrieval, following a previously published protocol.^55^ Subsequently, tumor sections were blocked with 3% BSA for 30 min and stained overnight at 4 °C using the following antibodies: anti-PD-L1 antibody (ab233482, 1:100, Abcam), anti-CD3 antibody (GTX 16669, 1:50, GeneTex, Irvine, CA, USA) or Anti-GrB antibody (14-8822-82, 1:200, eBioscience, San Diego, CA, USA). Afterward, the tumor sections were stained with Alexa Fluor 488-conjugated goat antirabbit (ab150077, 1:1000, abcam) for PD-L1 and CD3 or Alexa Fluor 488-conjugated goat antirat antibodies (ab150157, 1:1000, abcam) for GrB, respectively for 1 h at 25 °C. The resulting images were then captured using a fluorescence microscope (Axio Observer 7).

### Analysis of T Cells in TME

To analyze the population of T cells within the TME, cells were collected from the tumors on day 10 following various treatments. This was achieved using a tumor dissociation kit (Miltenyi Biotec, North Rhine-Westphalia, Germany) along with a dissociator (GentleMACS, Miltenyi Biotec) to obtain single-cell suspensions, which were then filtered through a 70 μm strainer (Smartstrainer, Miltenyi Biotec) to eliminate larger debris. Thereafter, the resulting single-cell suspensions underwent a blocking step with antimouse CD16/32 antibodies (101302, 1:25, BioLegend) and were subsequently stained with the following antibodies: APC/cyanine7- conjugated anti-CD45 (103116, 1:40, BioLegend), FITC-conjugated anti-CD3 (100204, 1:25, BioLegend), PE-conjugated anti-CD8a (100708, 1:100, BioLegend), and APC-conjugated anti-CD4 (100412, 1:100, BioLegend) antibodies. The antibody staining process occurred over a period of 30 min at 4 °C, followed by 7-AAD viability staining (420404, BioLegend). The stained cells were then analyzed using a cell sorter (Aurora CS, Cytek Biosciences, Fremont, CA, USA), and the data were analyzed using FlowJo Software (Treestar, Ashland, OR, USA).

### Analysis of Proinflammatory Cytokines in TME

For the analysis of pro-inflammatory cytokines, at the experimental end point (day 20), the tumor tissues were harvested and homogenized by RIPA lysis buffer containing a protease inhibitor cocktail with the assistance of the gentleMACS dissociator. The levels of pro-inflammatory cytokines (IFN-γ, IL-2, and TNF-α) in the homogenized tissues were subsequently determined using a multiplex assay (Bio-Plex Pro, Bio-Rad Laboratories).

### Determination of Levels of Blood Chemistry Parameters

To assess the biosafety of BMS202-loaded micelles, the blood samples were collected at the end of the experiment through heart puncture and then centrifuged to obtain the serum. The serum levels of ALT, AST, BUN, and creatinine were determined using a chemistry analyzer (Dri-Chem 4000i, Fujifilm, Tokyo, Japan).

### Statistical Analysis

All results are expressed as mean ± standard error. To identify differences between pairs of groups, an unpaired Student *t*-test was employed. A *P* value below 0.05 was defined as statistical significance.

## Supporting Information Available

^1^H NMR spectrum of mPEG–linker; cryo-EM image of blank micelles; immunoblotting images of PD-L1 expressed in 4T1 cells that had received various treatments; pharmacokinetics of C6 in different formulations after intravenous injection in tumor-free mice; fluorescence images of primary tumor sections stained for CD86; absolute counts of CD4^+^ and CD8^+^ T cells within 1 × 10^6^ CD45^+^ positive cells; relative body weight changes of test mice undergoing various treatments; H&E staining images showing major organs harvested from test mice following various treatments; serum levels of ALT, AST, BUN, and creatinine (PDF)
