# Supplementary material for: Immunomodulatory Prodrug Micelles Imitate Mild Heat Effects to Reshape Tumor Microenvironment for Enhanced Cancer Immunotherapy
Source: ACS Nano. 2024 Feb 12;18(7):5632–46. doi: 10.1021/acsnano.3c11186 (PMC10883120; doi:10.1021/acsnano.3c11186)
Supplement: Supplementary file 1 — nn3c11186_si_001.pdf [file nn3c11186_si_001.pdf]

Supporting Information

Immunomodulatory Prodrug Micelles Imitate Mild Heat Effects to Reshape Tumor Microenvironment for Enhanced Cancer Immunotherapy

Thi-Lan-Huong Ngo[a†], Kuan-Lin Wang[a,b†], Wen-Yu Pan[c], Ting Ruan[b], Yu-Jung Lin[a]**

[a] *Research Center for Applied Sciences, Academia Sinica, Taipei, 115201, Taiwan*

[b] *School of Medicine, College of Medicine, Fu Jen Catholic University, New Taipei City, 242062, Taiwan*

[c] *School of Medical Laboratory Science and Biotechnology, College of Medical Science and Technology, Taipei Medical University, Taipei, 110301, Taiwan*

[d] *Ph.D. Program in Medical Biotechnology, College of Medical Science and Technology, Taipei Medical University, Taipei, 110301, Taiwan*

*To whom correspondence should be addressed: 092810@mail.fju.edu.tw (T. Ruan) and linyujung@gate.sinica.edu.tw (Y. J. Lin).

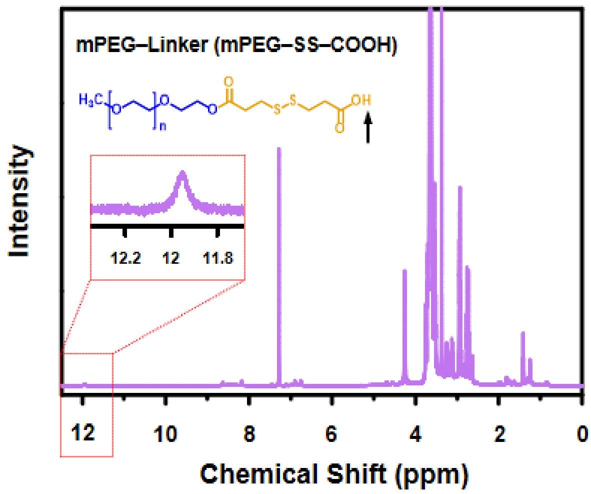

Figure S1. ^{1}H NMR spectrum of mPEG–linker (mPEG–SS–COOH).

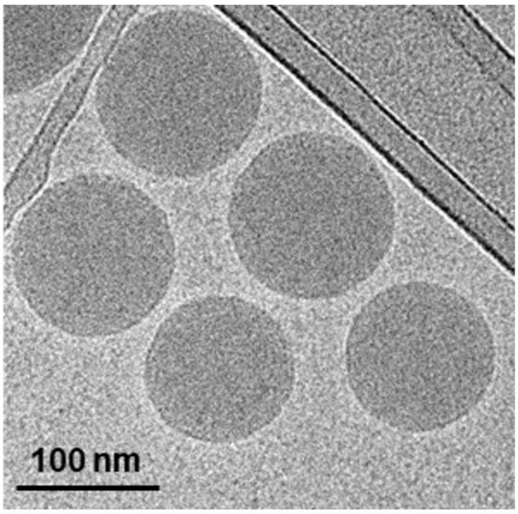

Figure S2. Cryo-EM image of blank micelles.

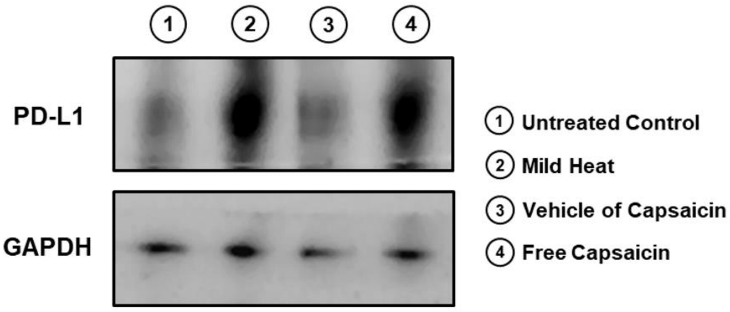

Figure S3. Immunoblotting images of PD-L1 expressed in 4T1 cells that had received various treatments.

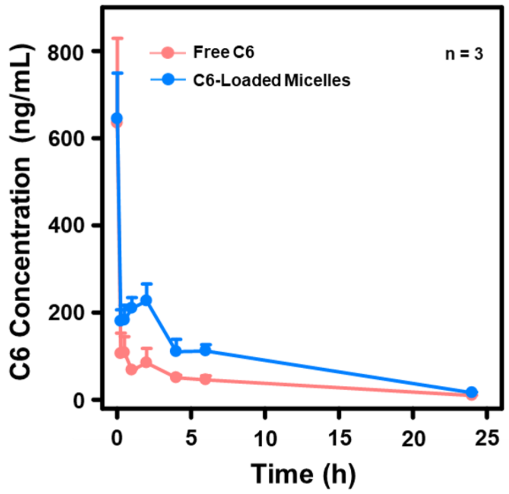

Figure S4. Pharmacokinetics of C6 in different formulations after intravenous injection in tumor-free mice.

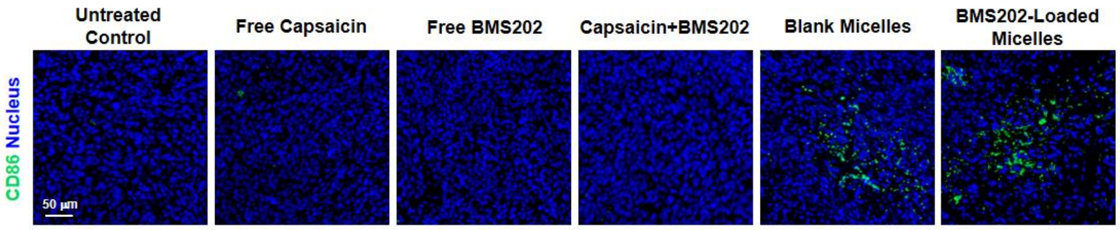

Figure S5. Fluorescence images of primary tumor sections stained for CD86.

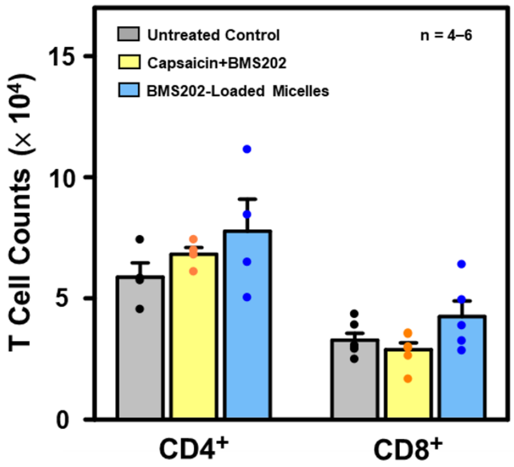

Figure S6. Absolute counts of $CD4^+$ and $CD8^+$ T cells within $1{\times}10^6$ $CD45^+$ positive cells.

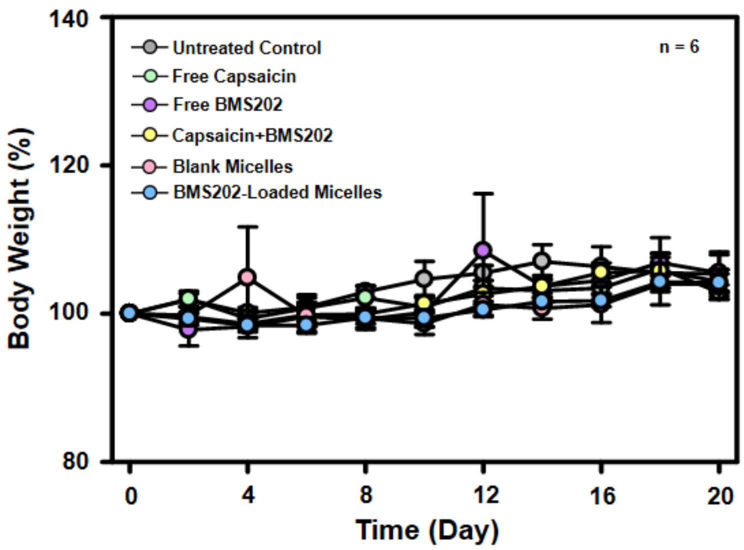

Figure S7. Relative body weight changes of test mice undergoing various treatments.

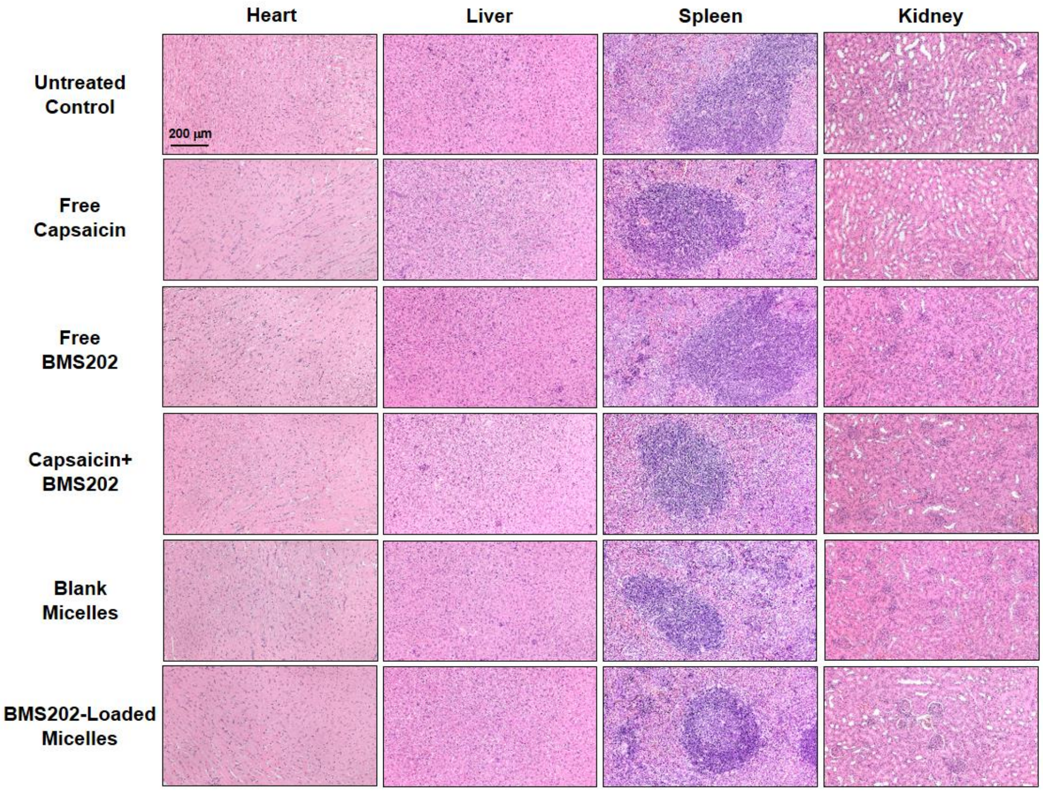

Figure S8. H&E staining images showing major organs harvested from test mice following various treatments.

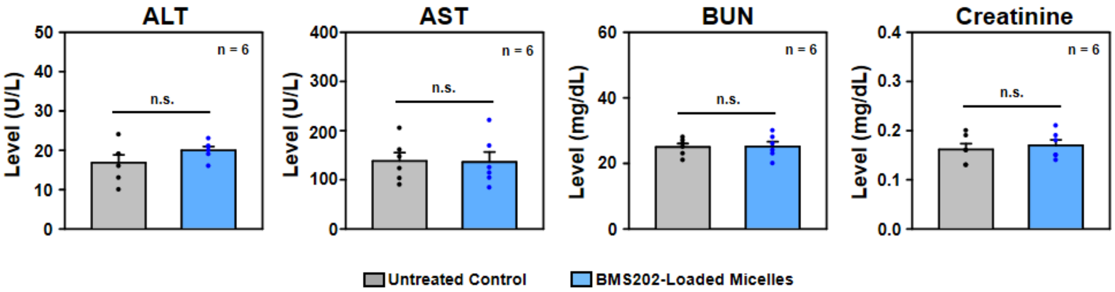

Figure S9. Serum levels of ALT, AST, BUN, and creatinine.
